# Giant Light Emission Enhancement in Strain-Engineered InSe/MS_2_ (M = Mo or W) van der Waals Heterostructures

**DOI:** 10.1021/acs.nanolett.4c04252

**Published:** 2025-02-05

**Authors:** Elena Blundo, Federico Tuzi, Marzia Cuccu, Michele Re Fiorentin, Giorgio Pettinari, Atanu Patra, Salvatore Cianci, Zakhar R. Kudrynskyi, Marco Felici, Takashi Taniguchi, Kenji Watanabe, Amalia Patanè, Maurizia Palummo, Antonio Polimeni

**Affiliations:** † Physics Department, Sapienza University of Rome, Piazzale Aldo Moro 5, 00185 Rome, Italy; ‡ Department of Applied Science and Technology, Politecnico di Torino, Corso Duca degli Abruzzi 24, 10129 Torino, Italy; § Institute for Photonics and Nanotechnologies, National Research Council, 00133 Rome, Italy; ∥ Faculty of Engineering, University of Nottingham, Nottingham NG7 2RD, U.K.; ⊥ Research Center for Materials Nanoarchitectonics, National Institute for Materials Science, 1-1 Namiki, Tsukuba 305-0044, Japan; # Research Center for Electronic and Optical Materials, National Institute for Materials Science, 1-1 Namiki, Tsukuba 305-0044, Japan; ∇ School of Physics and Astronomy, University of Nottingham, Nottingham NG7 2RD, U.K.; ○ INFN, Dipartimento di Fisica, Università di Roma Tor Vergata, Via della Ricerca Scientifica 1, 00133 Rome, Italy

**Keywords:** heterostructures, 2D materials, InSe, transition metal dichalcogenides, strain

## Abstract

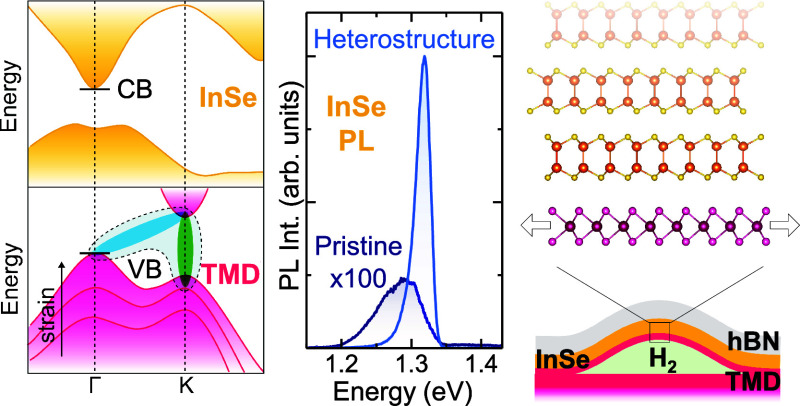

Two-dimensional (2D) heterostructures (HSs) offer unlimited possibilities for playing with layer number, order, and twist angle. The realization of high-performance optoelectronic devices, however, requires the achievement of specific band alignments, *k*-space matching between conduction and valence band extrema, and efficient charge transfer between the constituent layers. Fine-tuning mechanisms to design ideal HSs are lacking. Here, we show that layer-selective strain engineering can be exploited as an extra degree of freedom to tailor the band alignment and optical properties of 2D HSs. To that end, strain is selectively applied to MS_2_ (M = Mo or W) monolayers in InSe/MS_2_ HSs, triggering a giant photoluminescence enhancement of the highly tunable but weakly emitting InSe of up to >2 orders of magnitude. Resonant excitation measurements, supported by first-principles calculations, provide evidence of a strain-activated charge transfer from the MS_2_ monolayers toward InSe. The huge emission enhancement of InSe widens its range of applications for optoelectronics.

van der Waals (vdW) heterostructures (HSs) offer a vast playground for the realization of novel electronic and optoelectronic devices, due to the multitude of degrees of freedom they display, such as layer number, order, and twist angle. The weak vdW adhesion^1^ that keeps together different two-dimensional (2D) crystals is responsible for this unprecedented tunability, overcoming lattice mismatch issues and rotational constraints.^2^ This high tunability has been exploited to engender novel phenomena, such as superconductivity in twisted multilayer graphene,^3^ and to realize efficient electronic devices.^4−9^ However, the achievement of high-performance optoelectronic devices remains a challenge due to the necessity to find materials with specific band alignments, *k*-space matching conduction band minima (CBM) and valence band maxima (VBM), and efficient charge transfer between different layers.^10,11^ Fine-tuning mechanisms to design ideal HSs are still lacking. Strain has been used to shift the photoluminescence (PL) signal of HSs made of transition metal dichalcogenides (TMDs)^12^ and to modify the geometry of the moiré potential in twisted bilayers.^13^ In all cases, the entire HS was stretched.

Here, we propose a novel paradigm by selectively straining only one of the constituent materials of vdW HSs formed by MS_2_ (M = Mo or W) TMD monolayers (MLs) and InSe thin flakes, resulting in a giant enhancement in the emission efficiency of the latter.

The choice of InSe is grounded in the excellent properties it exhibits. Indeed, InSe features excellent transport properties, such as a high electron mobility^14,15^ (1 order of magnitude larger than for MoS_2_ and WS_2_ MLs^16,17^) and a quasi-direct and tunable optical bandgap (*E*_gap_), which makes it particularly appealing, *e.g.*, for fast photodetectors^18^ operating from the ultraviolet to the near-infrared range. Indeed, *E*_gap_ varies from ∼1.2 to ∼2.0 eV, going from bulk to two-layer (L) crystals (for MLs, the lowest-energy transition has an increased indirect character, and it is optically inactive for in-plane polarized light and only weakly coupled to z-polarized light).^15,19^

Research on InSe has rapidly developed, with the fabrication of HSs, such as graphene/InSe,^20^ p-InSe/n-In_2_O_3_,^21^ and n-InSe/p-GaSe,^22^ resulting in junctions with excellent transport characteristics. InSe/GaSe type II HSs were also exploited to create optically efficient interlayer excitons.^23^ The use of InSe for optoelectronic devices, however, is hampered by its relatively low radiative efficiency. As a matter of fact, while the CBM of InSe is located at Γ, the VB has a camel’s back shape, with the VBM slightly off the Γ point (see Figure 1a). For ≳6 layers, the VBM approaches Γ and InSe virtually becomes a direct gap semiconductor.^24^ However, the electric dipole orientation of InSe is perpendicular to the exfoliation planes, which leads to a poor coupling to light directed perpendicular to the InSe plane,^24−27^ namely, the geometry mainly employed in optical devices. Several works have reported on strategies to increase the optical efficiency of InSe, including bending of the flakes through pillars,^25^ ridges,^28^ and nanotexturing^29^ or the coupling of InSe with inorganic perovskites,^30^ but increases of factors of only 2–3 have been typically achieved.

**Figure 1:**
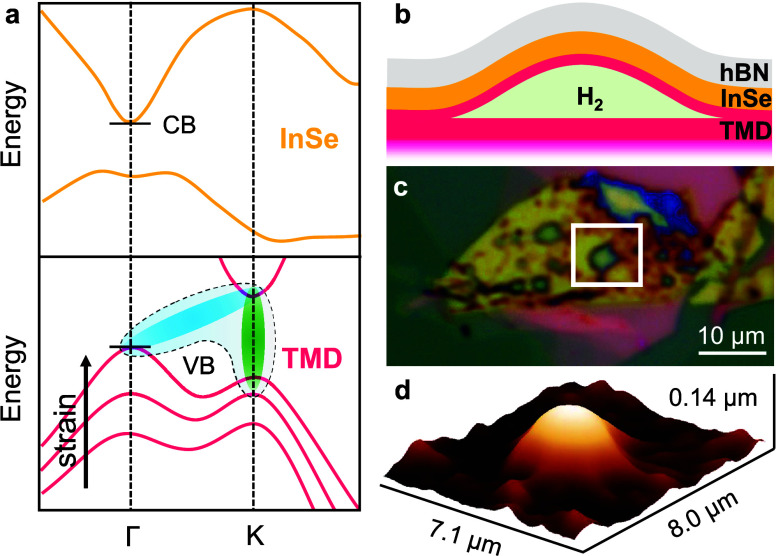
Heterostructured InSe/TMD bubbles. (a) Sketch of the band structure of few-layer-thick InSe and one-layer-thick TMDs. The effect of strain on the VB of TMDs is highlighted. For high strains, the valley at Γ goes above that at K and direct (green) and indirect (cyan) excitons hybridize. (b) Sketch of the system studied in this work, consisting of a heterostructured bubble. A few-layer-thick InSe flake is deposited atop a strained TMD ML in the shape of a bubble; hBN is used to cap the system. (c) Optical image of a flake with heterostructured bubbles. (d) AFM image of the bubble within the white rectangle in panel c.

In a previous work,^31^ InSe was coupled to multilayer (*N* ≥ 2) TMDs, whose VBM lies at the Γ point. A type II alignment was achieved in such HSs, with the observation of momentum-space direct (at Γ) interlayer excitons, formed by electrons at the InSe CBM and holes at the TMD VBM. Such a system was also exploited for light-emitting transistors.^32^ Although these HSs present the advantage of avoiding rotational constraints, their light emission is hindered by poor optical efficiency and significant sample-to-sample fluctuations. These issues can likely be ascribed to the intrinsically low radiative efficiency of InSe (as discussed above) and the *k*-space indirect nature of the bandgap in TMD multilayers. Additionally, these interlayer excitons have comparable intensity to intralayer excitons at cryogenic temperatures, but their spatially indirect nature makes their emission strongly decrease when increasing temperature.^31^

In this work, instead, InSe is coupled to a TMD (MS_2_) ML, whose optical bandgap is made indirect (Γ_VB_–K_CB_) by strain, yet, at variance with TMD multilayers, it is characterized by a remarkable oscillator strength thanks to the hybridization of direct and indirect exciton states^33,34^ (see Figure 1a). In fact, a fine-tuning of strain leads to a unique electronic configuration for the TMD ML in which excitons with an admixed direct–indirect character are observed.^34^ In turn, the strained MS_2_ ML retains a large light-to-charge conversion. Here, we show that the unique band structure configuration of the HS obtained by this approach, as evaluated by density functional theory (DFT) calculations, enables an efficient strain- and defect-assisted tunneling of photogenerated electrons and holes from the strained ML toward InSe, giving rise to a giant light emission enhancement of InSe.

To create HSs in which only the MS_2_ crystal is subject to high strain, while the InSe layer is not, we exploited strained WS_2_ and MoS_2_ MLs in the shape of microbubbles. The bubbles were created as described in ref (35). Bulk flakes are exposed to a low-energy ionized hydrogen beam; protons penetrate through the topmost layer, and molecular hydrogen forms and accumulates, leading to the formation of microbubbles on the flake surface. Such bubbles mainly have a thickness of just one layer^35,36^ and host sizable strains (up to >4%), whose extent increases from the edge toward the center.^1,37,38^ Optical spectroscopy and first-principles calculations revealed that the strain in the bubbles induces a direct-to-indirect transition^33^ [with the VBM shifting from K to Γ (see Figure 1a)] and that the nearly resonant direct and indirect excitons hybridize,^34^ leading to an efficient PL emission even when the indirect exciton is the lowest-energy state. These properties make strained TMDs promising systems for being coupled with InSe.

InSe and hBN flakes were mechanically exfoliated onto PDMS, and flakes with the desired thickness (∼6–10 L for InSe, <20 nm for hBN) were identified on the basis of their optical contrast. The InSe flakes were then deposited on some selected TMD bubbles; immediately thereafter, hBN was deposited atop the HS to prevent its oxidation^21^ (see Methods in the Supporting Information for details). A sketch of the final heterostructured bubble (HS bubble) is shown in Figure 1b, while the optical and three-dimensional atomic force microscope (AFM) images of a real sample are shown in panels c and d, respectively.

The optical properties of the HS bubbles were investigated by excitation with a 532 nm (2.33 eV) laser and by keeping the samples in vacuum to minimize sample oxidation. Micro-PL (μ-PL) measurements were performed from 5 K to room temperature (RT). Interestingly, the heterostructuring process has profound implications on the mechanics of the system in the low-temperature regime. In fact, hydrogen-filled TMD bubbles suddenly deflate at ∼30 K due to the gas-to-liquid phase transition of H_2_,^35,39^ while we do not observe any major change in the morphology of our HS bubbles even at 5 K (see Note 1 of the Supporting Information). As discussed therein, we attribute this to a tie-beam-like effect played by the InSe/hBN flakes, similar to that characteristic of tied-arch bridges or of Brunelleschi’s dome.^73^ Raman studies clearly demonstrate that the MS_2_ MLs are characterized by biaxial strains of ∼2% and that only a moderate strain reduction is observed with a temperature decrease from RT to 5 K (see Note 2 of the Supporting Information). On the contrary, PL measurements reveal that InSe is subject to minor strains of ∼0.05% (see Note 2 of the Supporting Information).

μ-PL measurements on single HS bubbles, at RT and low temperatures, for both MoS_2_ and WS_2_, reveal an efficient emission at ∼1.2–1.4 eV (see Figure 2a). This emission corresponds to the intralayer optical emission of InSe (either free or defect-localized^40^), but noticeably, its efficiency is much higher than that typically found in InSe flakes. Given the large spread in intensity (1–2 orders of magnitude) that characterizes InSe flakes with the same thickness,^41^ reliable information about the intensity of the HSs can be obtained by a direct comparison within the very same InSe flake. Therefore, in Figure 2, we compare the μ-PL signal recorded on the HS bubble (IN) and right outside (OUT), *i.e.*, where InSe is deposited on the bulk TMD flake and thus not in contact with the TMD ML bubble (see the sketch in panel b). Here, we note that the blue-shift of the IN InSe PL peak with respect to the OUT one apparent at 5 K excludes the possible formation of interlayer excitons and can instead be ascribed to an effective increase in the number of photogenerated carriers in the InSe layer. As we show below, these carriers are injected from the strained TMD ML, eventually leading to a saturation of the InSe lower-energy localized levels and hence to a shift of the PL toward the free exciton states. This scenario is supported by μ-PL studies as a function of *T* and by low-*T* power-dependent studies^40,42^ on both IN and OUT regions, as discussed in Note 3 of the Supporting Information. The InSe enhancement factor, defined as the ratio between the μ-PL peak intensities detected IN and OUT (*I*_IN_/*I*_OUT_, where *I* is the peak intensity), may show some variation with temperature, although an unequivocal trend cannot be found. Nevertheless, an indubitable emission enhancement between 1 and >2 orders of magnitude is systematically observed, as shown by a statistical analysis of several HS bubbles (see Figure 2c). It should be noted that the enhancement factors displayed in the figure were measured under nonresonant conditions, namely by excitation with a 532 nm laser. As shown below, even larger PL intensities can be detected by pumping in resonance with the TMD excitons.

**Figure 2:**
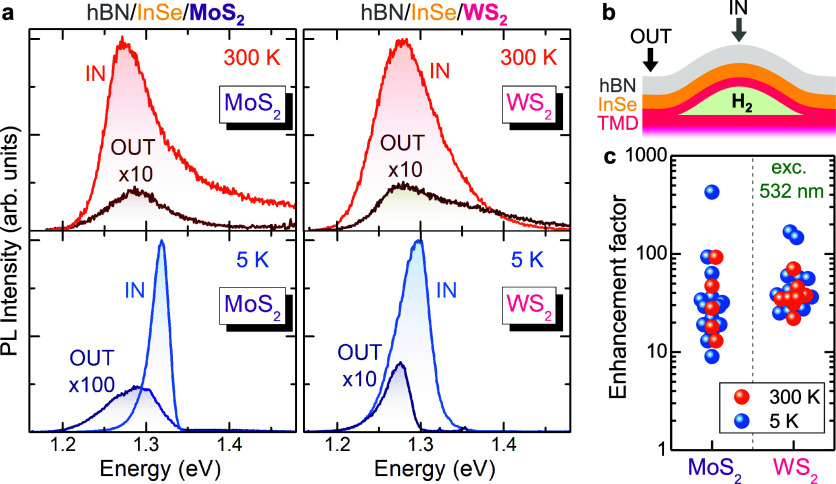
(a) Giant InSe emission enhancement in selectively strained InSe/MS_2_ heterostructures. (a) μ-PL spectra at 300 and 5 K of two HS bubbles (one with MoS_2_ as the TMD, left, and one with WS_2_, right) and of the region right outside the bubble, as indicated in the sketch in panel (b). (c) Summary of the ratios between the PL intensity in the HS bubbles and outside, measured in several MoS_2_- and WS_2_-based structures at 5 or at 300 K.

As discussed in Note 4 of the Supporting Information, we created several control samples to verify that there is no enhancement in the absence of strain and that the enhancement is due to strain solely and not to interference or exciton–dipole orientation effects. The giant PL enhancement of InSe in InSe/MS_2_ HS bubbles can thus be explained by hypothesizing a strain-induced charge transfer from the MS_2_ layer toward InSe. To ascertain this, we performed μ-PL excitation (μ-PLE) measurements on both hBN/InSe/MoS_2_ and hBN/InSe/WS_2_ HS bubbles (see Figure 3). Noticeably, clear exciton resonances are observed. For hBN/InSe/MoS_2_ HS bubbles, two resonances are found, at 1.84 and 2.01 eV. The former, attributed to the direct A exciton, is red-shifted by ∼0.1 eV with respect to the A exciton in planar MoS_2_ MLs due to strain.^33,43^ The second resonance at 2.01 eV is ∼0.17 eV above the lowest-energy one. Such a distance is compatible with the A–B exciton distance,^44^ and we thus ascribe the 2.01 eV resonance to the B exciton. In hBN/InSe/WS_2_ HS bubbles, instead, only one clear resonance, attributed to the A exciton, is observed at 1.98 eV, i.e., ∼0.1 eV below the A exciton in unstrained WS_2_ MLs (similar to the MoS_2_ case). μ-PLE measurements were also performed in a hBN/InSe/MoS_2_ unstrained HS. As shown in Note 5 of the Supporting Information, no resonances were found in the absence of strain. The μ-PLE measurements clearly point to a charge transfer from the MS_2_ ML to the InSe flake, which is activated by the strain selectively applied to the MS_2_ ML. As a matter of fact, a total strain of ∼0.5% applied to a MoS_2_/InSe HS as a whole resulted in no PL enhancement in ref (45). Therefore, our results clearly show that relatively high strains, applied to the MS_2_ ML, are required to activate a charge transfer. It should also be noted that such charge transfer is much more efficient than that previously obtained in type I 2D HSs (such as in MoTe_2_/WSe_2_ HSs, where only small PL enhancement factors of ≤2 orders of magnitude could be obtained^46^), which can be attributed to a nearly ideal condition achieved in our case by selective strain engineering.

**Figure 3:**
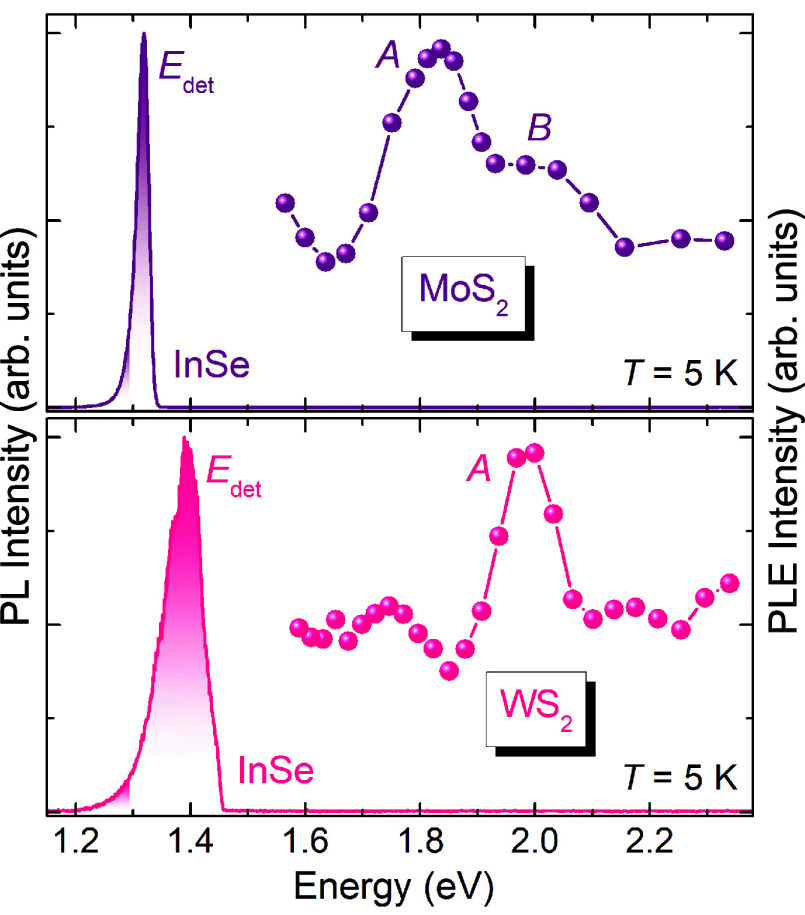
Energy-resonant photoexcited carrier transfer. PLE spectra of (a) a HS bubble based on MoS_2_ and (b) a HS bubble based on WS_2_. The corresponding PL band whose intensity was detected during the PLE measurements is displayed. Exciton resonances attributable to the A and B excitons of the TMD are highlighted.

To further elucidate the effect of strain on such HSs, we performed DFT calculations. The primary factor for ultrafast charge transfer in vdW HSs stems from the band alignment, coupled with the tendency of photoexcited electrons and holes to relax toward the CBM and VBM of the HS, respectively. In the case of type I band alignment, in which both CBM and VBM reside within the same material, like in the present system (see below), upon photoexcitation both electrons and holes migrate across the layers during relaxation. DFT simulations play a pivotal role in understanding charge transfer processes in such systems, providing valuable insights into the fundamental physics and guiding material design and optimization.

Focusing on InSe/MoS_2_ HSs, we investigated the electronic band alignment between the MoS_2_ ML and a 6L InSe slab. Because defect states, and especially sulfur vacancies, play an important role in determining the electronic properties of TMDs,^47^ we also took them into account by including a sulfur vacancy in the MoS_2_ ML in the simulation cell. The MoS_2_ and InSe bands were aligned to the vacuum level accounting for the interface dipole following the approach of refs (48) and (49). The band structures were computed with the Heyd–Scuseria–Ernzerhof range-separated hybrid functional HSE06^50^ (see Methods in the Supporting Information for further details). Panels a and c of Figure 4 show the band structure of the MoS_2_ ML at biaxial strains of 0% and 2%, respectively. Such a value was chosen to match the strain estimated through Raman experiments in Note 2 of the Supporting Information. In agreement with previous calculations,^51−56^ the application of 2% tensile biaxial strain shifts the VBM from K to Γ (inset of Figure 4c). In the CB, the minimum at the K point rapidly shifts in energy. On the contrary, the S vacancy introduces a pair of flat states within the bandgap, which are minimally affected by strain. It also induces an additional defect state within the VB of MoS_2_, in agreement with previous calculations and experiments.^57−59^ Lying below the VBM, this level does not impact the proposed charge transfer mechanism and is not shown in Figure 4. The band structure of 6L InSe is displayed in Figure 4b to be readily compared with the MoS_2_ unstrained and strained cases.

**Figure 4:**
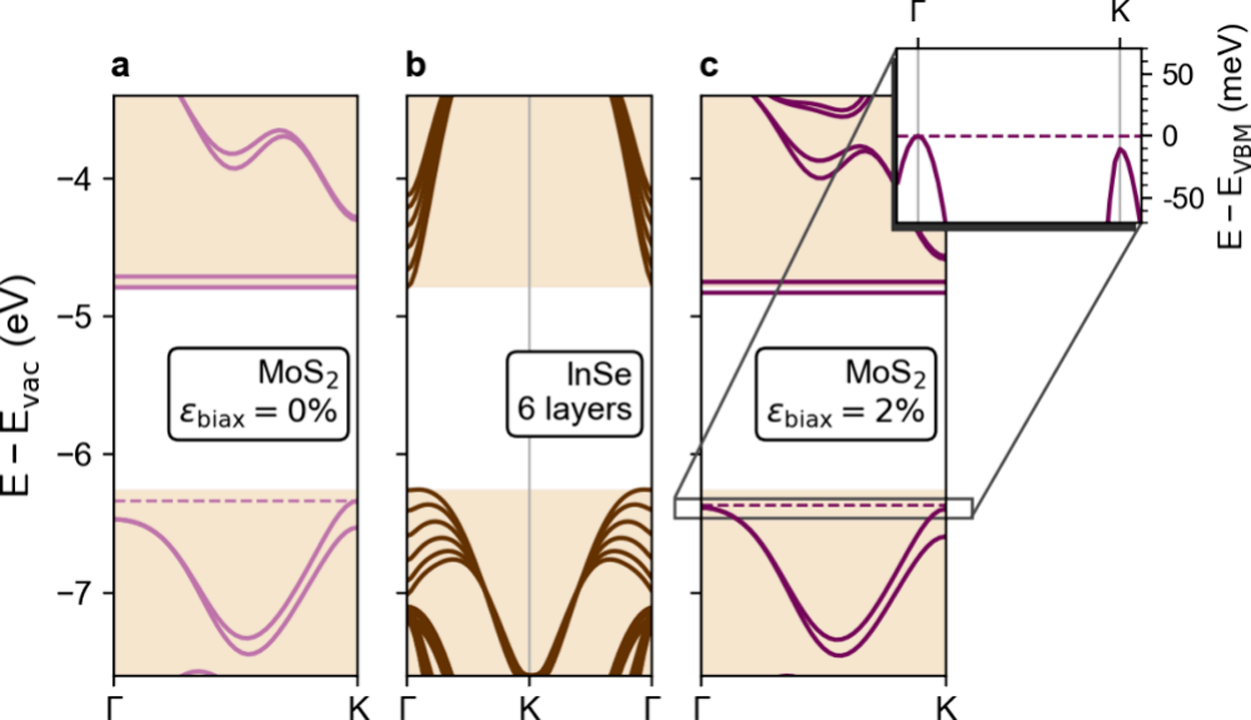
Heterostructured bubble band alignment. DFT-calculated band structures of (a) a MoS_2_ ML with S vacancies at 0% strain, (b) a 6L InSe slab, and (c) a MoS_2_ ML at 2% biaxial strain. The shaded regions mark the VB and CB of 6L InSe in all panels. The inset of panel c shows a close-up of the top VB of strained MoS_2_ at the Γ and K points.

The computed band structures provide the electronic scenario accounting for the giant PL enhancement of InSe experimentally observed in the HS bubbles. A type I band alignment is found in the absence and presence of strain, and the TMD and InSe VBs feature a sizable overlap around the Γ point (see Figure 4). When they are in contact, charge will equilibrate between the two materials, with the CBM states possibly being partly occupied due to native n-doping, as often experimentally observed.^60,61^

Light is strongly absorbed by the A exciton of MoS_2_, mainly due to photoexcited electrons and holes at the K point in the Brillouin zone (BZ). When the TMD ML is unstrained, electrons and holes recombine radiatively at the K point on the picosecond scale^62^ and no carrier transfer toward InSe occurs. In contrast, the VBM upshift introduced by tensile strain allows the fast phonon-mediated relaxation of holes from K to Γ, which takes place on the femtosecond scale.^63^ In turn, the *k*-space indirect character of the exciton in strained MoS_2_ considerably slows its recombination (up to a few nanoseconds^33^). Then, holes in the strained MoS_2_ ML can tunnel to InSe at those points in the BZ around Γ, where the bands of the two materials cross and tunneling can efficiently occur without energy or momentum exchange.^64,65^ The injected holes radiatively recombine in InSe. Charge is then rebalanced by the relaxation of the photogenerated electron in MoS_2_ and the refilling of electrons in the InSe CBM from the TMD ML. Indeed, due to the lack of a fast recombination pathway in the strained TMD ML, electrons in MoS_2_ can tunnel to the CBM of InSe, albeit on a time scale longer than that of hole transfer, due to the different *k*-space points of the CB minima in InSe and MoS_2_.^45^ In this respect, the presence of sulfur vacancies in the TMD ML can contribute to increase the efficiency of the electron transfer to InSe. As shown in Figure 4, the defect levels close to the MoS_2_ CBM overlap in energy with the CBM of InSe. Electrons populating these states can efficiently tunnel to the InSe CBM, thanks to their *k*-space delocalization that allows for tunneling with relaxed *k*-conservation transfer. Therefore, the density of sulfur vacancies in the TMD monolayer can be an influencing factor in the efficiency of this process, potentially accounting for the variations in the enhancement factor observed in Figure 2b and offering a further degree of control.

Additional defects in InSe, such as Se vacancies, have not been explicitly included in the simulation because they induce localized electronic states below the CBM or above the VBM, which can be readily saturated by the injected carriers.^66−68^ The band alignment calculated at the HSE level, in Figure 4, is confirmed by the results obtained from the explicit simulation of the full 6L-InSe/ML-MoS_2_ interface, with the Perdew–Burke–Ernzerhof exchange-correlation functional, reported in Note 6 of the Supporting Information. Hence, the concurrent injection of electrons and holes from the strained TMD ML (where efficient light absorption takes place) can populate the band edges of InSe and shift the PL mechanism to more efficient free exciton radiative recombination.

Moreover, we highlight that, for an increasing number of InSe layers, the bandgap slightly decreases (by ∼0.1 eV in going from 6 to 10 layers), so that the results of the calculations performed for 6L InSe can be generalized to *N*L InSe with 6 ≤ *N* ≤ 10 (used in the experiments). A mechanism analogous to that described for InSe/MoS_2_ HS bubbles is also expected for WS_2_-based HSs. In that case, we remark that while the CBM of WS_2_ lies above the CBM of MoS_2_ (by ∼0.3 eV^69^), S vacancies in WS_2_ monolayers give rise to deeper defect states, emitting ∼0.5 eV below the neutral exciton.^70^ In turn, the WS_2_ defect band is expected to lie very close in energy to that of MoS_2_ and thus be quasi-resonant with the InSe CBM. Finally, we remark that strain in InSe could affect its optical properties^71,72^ but would not affect the proposed charge transfer mechanism, as demonstrated by DFT calculations discussed in Note 7 of the Supporting Information.

In this work, we developed a novel paradigm to engineer the optoelectronic properties of 2D HSs by demonstrating how layer-selective stretching can be efficaciously used to tailor the electronic properties of the system. Layer-selective strains can be generally achieved by deposition of 2D flakes on prestretched 2D materials. Here, we specifically investigated the coupling of 6–10-layer-thick InSe with (strained) MS_2_ ML bubbles (M = Mo or W). Strain was shown to be responsible for a giant PL enhancement of the InSe signal between 1 and >2 orders of magnitude, at both cryogenic and room temperature. PLE measurements clearly proved that strain activates electronic coupling between the HS constituent layers, entailing a charge transfer from the TMD to InSe. DFT calculations confirm that a type I alignment is obtained and highlight the possible mechanisms responsible for the PL enhancement. This can be attributed to a strain-induced K-to-Γ VBM crossover along with the presence of S vacancy states near the CBM in MS_2_, leading to an efficient tunneling of holes (in the vicinity of Γ) and electrons (through momentum-delocalized defect states) at those points in the BZ where electronic states overlap in energy and momentum. In turn, a 2D type I HS characterized by an unprecedentedly efficient charge transfer, much larger than in previous TMD-based HSs, is achieved, thanks to the nearly ideal band alignment triggered by selective strain engineering. The significant enhancement of the PL efficiency of InSe achieved here, paired with its highly remarkable electronic and transport properties, dramatically improves the prospects for the exploitation of this material in a wide range of optoelectronic applications.

## Supporting Information Available

Methods; further morphological, Raman, and PL studies; and further calculations and information about the theoretical model (PDF)
