# Supplementary material for: Giant Light Emission Enhancement in Strain-Engineered InSe/MS2 (M = Mo or W) van der Waals Heterostructures
Source: Nano Lett. 2025 Feb 5;25(9):3375–82. doi: 10.1021/acs.nanolett.4c04252 (PMC11887447; doi:10.1021/acs.nanolett.4c04252)
Supplement: Supplementary file 1 — nl4c04252_si_001.pdf [file nl4c04252_si_001.pdf]

SUPPORTING INFORMATION for

Giant light emission enhancement in strain-engineered InSe/MS_2 (M=Mo,W) van der Waals heterostructures

Elena Blundo,[1,*] Federico Tuzi,[1] Marzia Cuccu,[1] Michele Re Fiorentin,[2] Giorgio Pettinari,[3] Atanu Patra,[1] Salvatore Cianci,[1] Zakhar R. Kudrynskyi,[4] Marco Felici,[1] Takashi Taniguchi,[5] Kenji Watanabe,[6] Amalia Patanè,[7] Maurizia Palummo,[8] and Antonio Polimeni[1,*]

[1] *Physics Department, Sapienza University of Rome, Piazzale Aldo Moro 5, 00185 Rome, Italy.*
[2] *Department of Applied Science and Technology, Politecnico di Torino, corso Duca degli Abruzzi 24, 10129 Torino, Italy*
[3] *Institute for Photonics and Nanotechnologies, National Research Council, 00133 Rome, Italy.*
[4] *Faculty of Engineering, University of Nottingham, Nottingham, NG7 2RD, UK.*
[5] *Research Center for Materials Nanoarchitectonics, National Institute for Materials Science, 1-1 Namiki, Tsukuba 305-0044, Japan.*
[6] *Research Center for Electronic and Optical Materials, National Institute for Materials Science, 1-1 Namiki, Tsukuba 305-0044, Japan.*
[7] *School of Physics and Astronomy, University of Nottingham, Nottingham, NG7 2RD, UK.*
[8] *INFN, Dipartimento di Fisica, Universitá di Roma Tor Vergata, Via della Ricerca Scientifica 1, 00133 Rome, Italy.*

* Corresponding authors: antonio.polimeni@uniroma1.it, elena.blundo@uniroma1.it

Contents

Methods **1**

Supporting Note 1. Heterostructured bubble morphology *vs* temperature **3**

Supporting Note 2. Strain characterisation in heterostructured bubbles **5**

2.1 Strain estimation for the TMD 5

2.2 Strain estimation for InSe 8

Supporting Note 3. Photoluminescence studies of heterostructured bubbles *vs* temperature **10**

3.1 PL *vs* T 10

3.2 PL *vs* P_{exc} 11

Supporting Note 4. Photoluminescence studies of several control samples **13**

Supporting Note 5. Photoluminescence excitation studies of an unstrained hBN/InSe/MoS_2 heterostructure **15**

Supporting Note 6. DFT simulations of explicit InSe/MoS_2 interfaces. **16**

Supporting Note 7. DFT simulations of strained InSe. **18**

References **19**

Methods

Sample fabrication

The HSs were fabricated by the standard dry transfer technique. TMD flakes were mechanically exfoliated by the scotch tape method and deposited on SiO_2/Si substrates. The samples were then loaded into a Kaufman chamber and exposed to hydrogen-ion atoms with energies of 10-20 eV, according to the procedure discussed in refs. [1, 2], leading to the formation of bubbles. In particular, in our hydrogenation process, we irradiate the samples with H-ions (namely, protons and ionised H_2^+ molecules in a percentage of about 3% and 97%, respectively [3]) of which near exclusively protons penetrate through the first top layers due to their smaller size as compared to H_2^+ (TMDs were shown to be H_2-impermeable [4]). The accelerated protons penetrating through the flake surface interact with electrons provided by the ground contact and in turn form (neutral) H_2 molecules, just one layer beneath the surface. There, (neutral) H_2 molecules become trapped and form bubbles, as we reported for the first time in Ref. [1]. The large size of the hydrogen molecules as compared to the crystal inter-spacing distances does not permit H_2 to diffuse through the TMD layers [5] and the formed bubbles last up to >5 years according to our observations. We finally remark that *neutral* molecules form and thus no charge carrier trapping should be expected.

Atomic force microscopy

AFM measurements were performed using a Veeco Digital Instruments Dimension D3100 microscope equipped with a Nanoscope IIIa controller, employing Tapping Mode monolithic silicon probes with a nominal tip curvature radius of $5 - 10\,\mathrm{nm}$ and a force constant of $40\,\mathrm{N\,m^{-1}}$. All the scans were performed at room temperature and at ambient conditions. All the data were analysed with the Gwyddion software.

μ-PL measurements

For μ-PL measurements, the samples were placed in a closed-circuit He cryostat by Montana Instruments; vacuum conditions were established (chamber pressure < 2 mTorr) and the sample was brought to the desired temperature (or kept at RT). The excitation laser was provided by a single frequency Nd:YVO_4 laser (DPSS series by Lasos) emitting at 532 nm. To avoid oxidation effects, we employed low laser powers, typically equal to 4 μW (and always below 40 μW). The luminescence signal was spectrally dispersed by a 20.3 cm focal length Isoplane 160 monochromator (Princeton Instruments) equipped with a 150 grooves/mm and a 300 grooves/mm grating and detected by a back-illuminated N_2-cooled Si CCD camera (100BRX by Princeton Instruments). The laser light was filtered out by a very sharp long-pass Razor edge filter (Semrock). A 100× long-working-distance Zeiss objective with NA = 0.75 was employed to excite and collect the light, in a backscattering configuration and using a confocal setup.

μ-PL excitation measurements

For μ-PL excitation (μ-PLE), we employed the same ps supercontinuum laser used for tr μ-PL. The laser wavelength was automatically changed by an acousto-optic tunable filter and employing a series of shortpass and longpass filters to remove spurious signals from the laser. The detection wavelength was selected using the same monochromator and detector employed for cw μ-PL measurements.

μ-Raman measurements

μ-Raman measurements were taken in the same experimental configuration employed for μ-PL measurements. In this case, the Raman signal was spectrally dispersed by a 75 cm focal length Acton monochromator (Princeton Instruments) equipped with a 12000 grooves/mm grating.

Density functional theory calculations
Simulations were performed with DFT using the plane-wave expansion method as implemented in the Quantum ESPRESSO package [6, 7].

Fully relativistic, norm-conserving pseudopotentials [8] were employed to account for spin-orbit coupling. Convergence was achieved considering an 80 Ry kinetic energy cutoff and an $8 \times 8 \times 1$ ($9 \times 9 \times 1$) Monkhorst-Pack k-point mesh for the 6L-InSe slab (TMD monolayer). The k-point meshes were accordingly rescaled in supercell calculations. The optimised geometries of TMD MLs and 6L-InSe were obtained by performing structure relaxations with the Perdew-Burke-Ernzerhof (PBE) functional [9]. A 20 Å vacuum region along the direction perpendicular to the slab plane was introduced to ensure decoupling of periodic replicas. Structure relaxations were assumed to have reached convergence when the maximum component of the residual ionic forces was smaller than 10^{-8} Ry/Bohr. The sulphur vacancy in the TMD monolayer was simulated considering a 5×5 supercell.

The Heyd–Scuseria–Ernzerhof range-separated hybrid functional (HSE06) [10] was chosen over PBE to calculate the electronic bandstructures and band alignments due to its superior capability in accurately describing electronic properties. While the PBE functional is commonly employed for structure optimisations owing to its computational efficiency, it frequently produces underestimated bandgaps, which can lead to incorrect predictions of band alignments. The HSE06 functional represents a reasonable and practical choice to investigate charge transfer and electronic properties, providing accurate results at a lower computational cost compared to more advanced methods like GW calculations.

The band alignment in HSs is affected by dipoles originating from the atomic configuration and orientation of the two contacting materials. To account for the interface dipole between the TMD and InSe, we adopted the methodology outlined in Ref. [11], which adjusts the initial alignment derived from isolated phases by considering the shift in the inner potential between the two phases in a model, lattice-matched, interface. However, while advantageous for their reduced number of atoms, lattice-matched interfaces inherently introduce undesired strain in the two phases. This is then compensated for by considering the potential offset between the strained and unstrained lattices.

Additional defects in InSe, such as Se-vacancies, have not been explicitly included in the simulation since they induce localised electronic states below the CBM or above the VBM, that can be readily saturated by the injected carriers [12].

Finally, to verify the predictions and prove the type-I band-alignment of the real interface, we simulated supercells of the explicit ML-MoS_2/6L-InSe systems. The interfaces between 6L-InSe and 0%- and 2%-strained TMD MLs were obtained with the CellMatch code [13] (see Supporting Note 6) and are reported in Supporting Figure 6.1. Due to the large number of electrons (3504 and 3224 electrons for the unstrained and strained HS, respectively), the geometry optimisation and the analysis of the electronic properties were performed with the rev-vdW-DF2 [14] GGA functional, without spin-orbit coupling.

Supporting Note 1. Heterostructured bubble morphology *vs* temperature

The heterostructuring process employed in this work —consisting in the deposition of thin InSe and hBN flakes on transition metal dichalcogenide (TMD) bubbles— has profound implications on the mechanics of the system in the low temperature regime. In fact, hydrogen-filled TMD bubbles are known to suddenly deflate at about 30 K due to the gas-to-liquid phase transition of H_2 [1, 15], while we do not observe any major change in the morphology of our heterostructured bubbles (HS-bubbles) even at 5 K. This is shown, as an example, for two hBN/InSe/WS_2 HS-bubbles in Fig. 1.1: Figs. 1.1**a** and **b** show the optical images of a flake containing the two HS-bubbles, taken at 6 K and room-temperature (RT), respectively. The two HS-bubbles are highlighted by white circles. The yellow circle, instead, highlights the position of a WS_2 bubble which was not covered neither by InSe nor by hBN. Indeed, the two HS-bubbles are clearly visible in both images, while the non-capped bubble is not visible in the image acquired at 6 K since it deflated at around 30 K with the occurring of the gas-to-liquid transition of the hydrogen inside the bubble. The same bubble can in fact be seen at RT, as it can be noticed by looking at the optical image shown in panel **b**. Finally, Fig. 1.1**c** shows an atomic force microscope (AFM) image of the same flake, at RT.

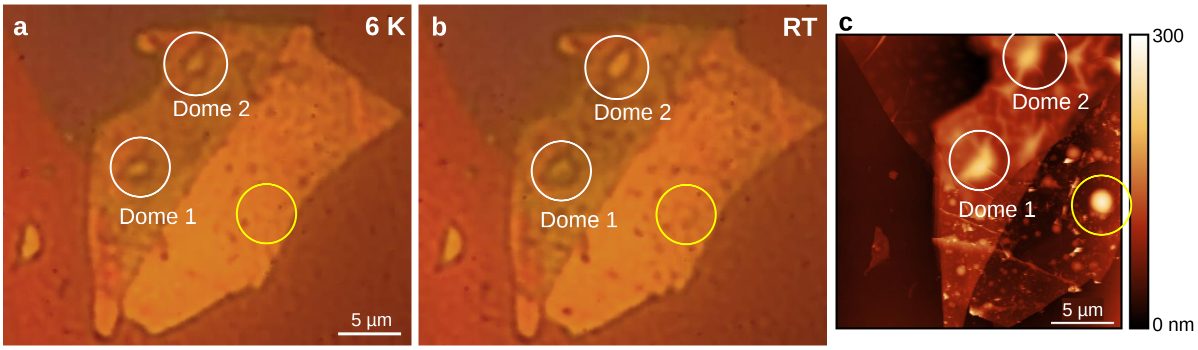

Figure 1.1: Morphology of heterostructured bubbles *vs* temperature. **a** Optical image acquired at 6 K of two hBN/InSe/WS_2 HS-bubbles, highlighted by the white circles. The yellow circle highlights the position of a WS_2 bubble which was not covered neither by InSe nor by hBN. **b** Same as **a** but at RT. **c** AFM image of the same flake of panels **a** and **b**, acquired at RT.

Noticeably, the two HS-bubbles show only a moderate size variation between RT and 6 K. We attribute this effect to the role played by the InSe and hBN flakes, that adapt to the bubble underneath during the deposition process, minimising the total energy of the system. The deflation of the bubble when H_2 liquefies would, in turn, imply a mechanical deformation of the above flakes and thus an energy cost. The cost of keeping the bubble in-shape is likely lower, leading to this peculiar effect for which the strain is maintained even below 30 K, when no internal pressure is exerted by hydrogen on the TMD ML. Raman studies, discussed in Supporting Note 2, corroborate this finding by demonstrating that only a moderate strain reduction is observed when decreasing T from RT to 5 K.

This mechanical effect is similar to that played by tied arches in bridges, see Fig. 1.2. In tied-arch bridges (HS-bubbles), the main beam (TMD dome) is coupled to the arch (InSe+hBN) via suspenders (van der Waals forces). The rigidity of the arch (InSe+hBN) and strength of the suspenders (van der Waals adhesion) make it so that the whole structure maintains its shape even when the main beam (TMD dome) is subject to external forces, *e.g.* the gravity and when heavy vehicles pass through the bridge (the hydrogen gas undergoes a gas-to-liquid transition).

The mechanical effect observed of our micro-domes represented by the HS-bubbles also resembles that of the giant (external diameter = 54.8 m) and beautiful Brunelleschi's Dome in

Florence. As sketched in Fig. 1.2**b**, that Dome is characterised by a double-layer structure, which makes the Dome structure robust, similarly to our HS-micro-domes.

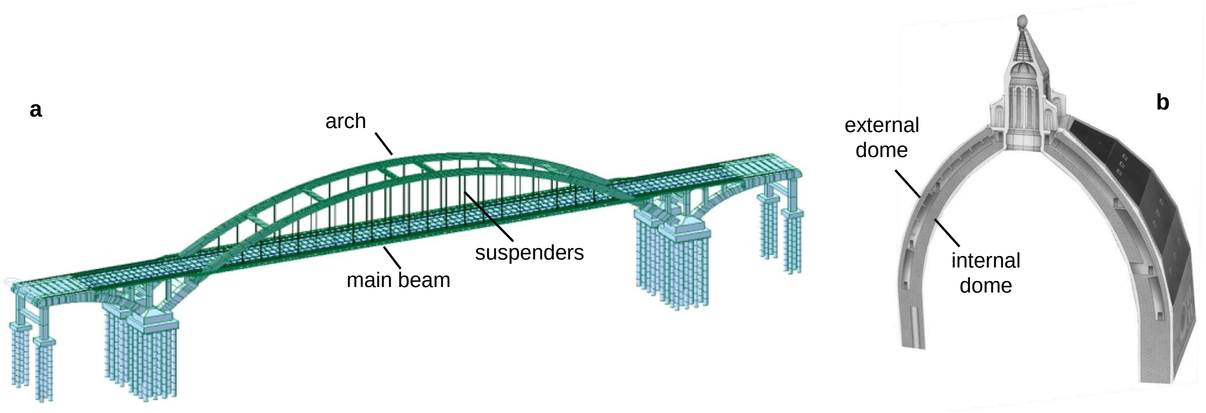

Figure 1.2: Tie-beam-like effect, the analogy with bridges and brick domes. a Sketch of a tied-arch bridge. Adapted with permission from J. Sun *et al.*, Int. J. Steel Struct. **23**, 191–207 (2023) [16]. Copyright 2024 Springer Nature. **b** Schematic image of the double-dome structure of Brunelleschi's Dome (in Florence, Italy).

Supporting Note 2. Strain characterisation in heterostructured bubbles

2.1 Strain estimation for the TMD

We investigated the structural properties of the HS-bubbles by performing Raman measurements as a function of T on hBN/InSe/MoS_2 bubbles. Specifically, we monitored the Raman signal of MoS_2 in the phonon regions of the in-plane E^1_{2g} and of the out-of-plane A_{1g} modes. Fig. 2.1**a** shows the micro-Raman spectra acquired on a hBN/InSe/MoS_2 bubble as a function of temperature. The Raman spectra show the appearance of both the E^1_{2g} and A_{1g} modes of the bubble and those of the bulk flake beneath the bubble (with the bubble peak being at lower frequency than the bulk one, for both the modes). The bulk peaks feature a moderate thermal shift with temperature. The bubble peaks feature a larger shift, suggesting a strain variation, as testified by the quantitative analysis displayed in panel **b**, where the Raman shift values ($\Delta\tilde{\nu}$) of all modes are shown.

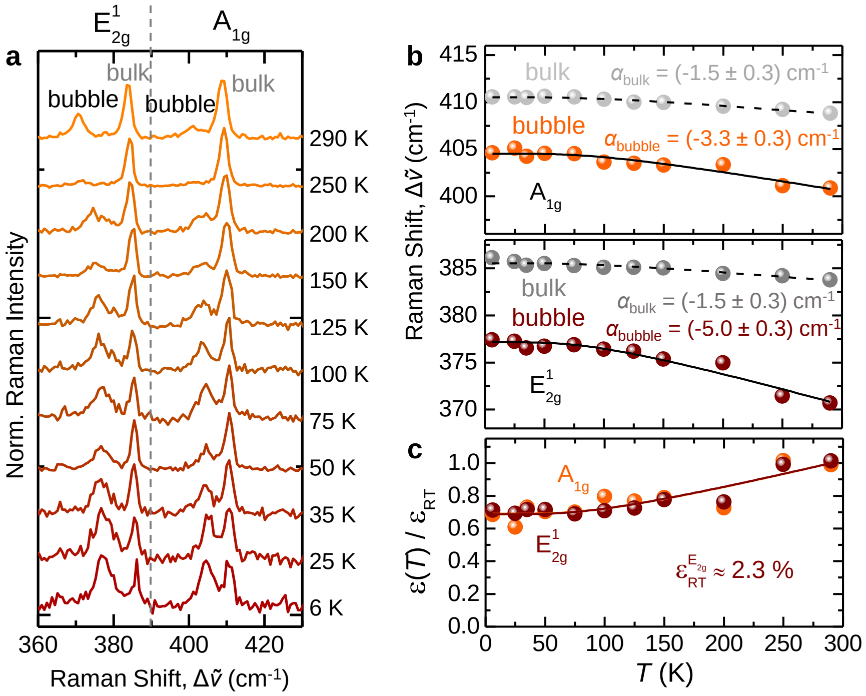

Figure 2.1: Structural properties of a heterostructured bubble *vs* temperature investigated by Raman spectroscopy. a Raman spectra of a hBN/InSe/MoS_2 HS-bubble acquired in the phonon regions of the MoS_2 in-plane E^1_{2g} and of the out-of-plane A_{1g} modes at different temperatures, stacked by y-offset. The Raman modes of both the bubble and the bulk beneath the bubble can be seen. **b** Raman shifts of the A_{1g} and E^1_{2g} modes of both the bubble and the bulk *vs* T. The solid (dashed) lines are fits to the data concerning the bubble (bulk) via Eq. 2.1. The α constants obtained by fitting the data are displayed. **c** Strain variation with temperature calculated starting from the experimental Raman shifts shown in panel **b** with respect to the room-temperature (RT, corresponding to 290 K) value, estimated following Eq. 2.2 as: $\varepsilon(T)/\varepsilon_{\mathrm{RT}} = [\Delta\tilde{\nu}^{\mathrm{ML}}(T) - \Delta\tilde{\nu}^{\mathrm{bubble}}(T)]/[\Delta\tilde{\nu}^{\mathrm{ML}}(\mathrm{RT}) - \Delta\tilde{\nu}^{\mathrm{bubble}}(\mathrm{RT})]$. The solid lines display the strain calculated with the same approach, but starting from the fitting curves displayed in panel **b**.

The Raman shift values were fitted with the formula

$$\Delta\tilde{\nu}\,(T) = \Delta\tilde{\nu}_0 + \alpha\left(1 + \frac{2}{e^{\frac{hc\Delta\tilde{\nu}_0}{2k_{\mathrm{B}}T}} - 1}\right) \tag{2.1}$$

where $\Delta\tilde{\nu}_0$ and A are constants (left as fitting parameters) and only the lower-order three-phonon processes of the anharmonic Klemens model are taken into account via the parameter α [17, 18, 15] (h is the Planck constant, c is the speed of light, and k_{B} is the Boltzmann constant).

The Raman mode frequency of the reference unstrained monolayer (ML), $\Delta\tilde{\nu}^{\mathrm{ML}}(T)$, can be obtained from the measured bulk mode by just adding (subtracting) a small 1.5 cm^{-1} rigid shift to the $\mathrm{E_{2g}}$ ($\mathrm{A_{1g}}$) peak [19]. Therefore, from the frequency position of the Raman peaks associated to the bulk and to the bubble modes, information on the biaxial strain $\varepsilon_{\mathrm{biax}}$ at different temperatures can be extracted quantitatively by applying the following formula:

$$\varepsilon_{\mathrm{biax}}(T) = \frac{1}{2}\left[\frac{\Delta\tilde{\nu}^{\mathrm{ML}}(T) - \Delta\tilde{\nu}^{\mathrm{bubble}}(T)}{\frac{d\Delta\tilde{\nu}}{d\varepsilon_{\mathrm{tot}}}}\right] \tag{2.2}$$

where $\frac{d\Delta\tilde{\nu}}{d\varepsilon_{\mathrm{tot}}}$ is the shift rate of the Raman mode with (total) strain (assumed to be constant at all temperatures [20]) and the total strain is divided by 2 to obtain the biaxial strain, *i.e.* the strain induced along just one direction (*i.e.*, the radial or the circumferential one, which are equivalent at the top of the bubble [19]). Fig. 2.1**c** shows the strain variation with temperature, calculated as the strain at temperature T normalised to the strain at room-temperature (RT). A quantitative estimate of the in-plane biaxial strain can be obtained by the knowledge of the shift rate of the in-plane Raman mode, which is equal to (3.24 ± 0.41) $\mathrm{cm}^{-1}/\%$ [19]. In turn, a biaxial strain of about 2.3% is estimated at RT, with $\sim 30\%$ decrease at cryogenic temperatures (*i.e.*, $\varepsilon_{\mathrm{biax}} \approx 1.6\%$).

We repeated a similar analysis for a second hBN/InSe/MoS_2 bubble, as shown in Fig. 2.2. The results found in this second case are consistent with those found for the previous bubble. In this case, a RT biaxial strain equal to about 2.2% is estimated, with a $\sim 30-40\%$ decrease at cryogenic temperatures (*i.e.*, $\varepsilon_{\mathrm{biax}} \approx 1.3-1.5\%$).

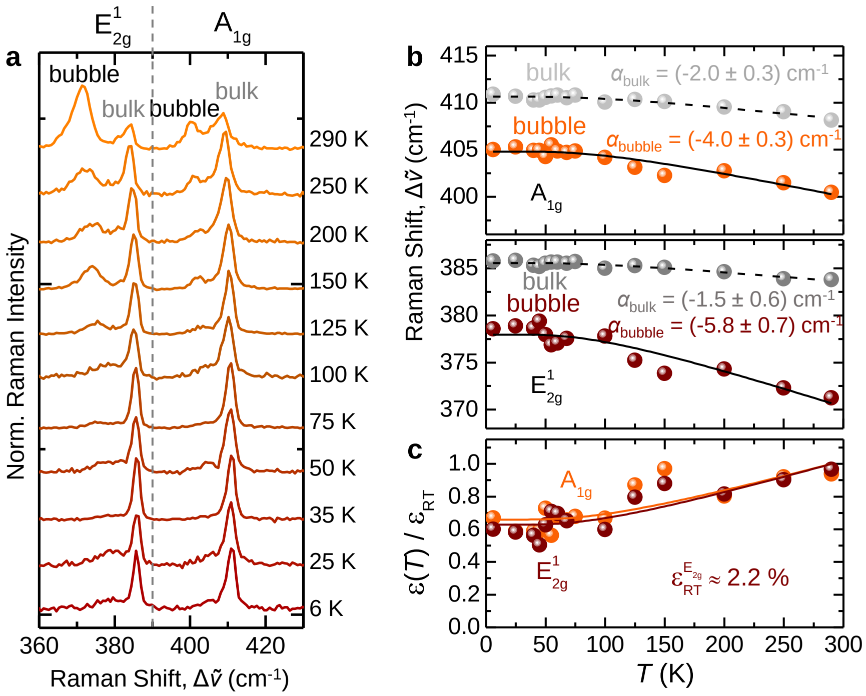

Figure 2.2: Structural properties of another heterostructured bubble *vs* temperature investigated by Raman spectroscopy. a Raman spectra of another hBN/InSe/MoS_2 HS-bubble acquired in the phonon regions of the MoS_2 in-plane E^1_{2g} and of the out-of-plane A_{1g} modes at different temperatures, stacked by y-offset. The Raman modes of both the bubble and the bulk beneath the bubble can be seen. **b** Raman shifts of the A_{1g} and E^1_{2g} modes of both the bubble and the bulk *vs* T. The solid (dashed) lines are fits to the data concerning the bubble (bulk) via Eq. 2.1. The α constants obtained by fitting the data are displayed. **c** Strain variation with temperature calculated starting from the experimental Raman shifts shown in panel **b** with respect to the room-temperature (RT, corresponding to 290 K) value, estimated following Eq. 2.2 as: $\varepsilon(T)/\varepsilon_{\mathrm{RT}} = [\Delta\tilde{\nu}^{\mathrm{ML}}(T) - \Delta\tilde{\nu}^{\mathrm{bubble}}(T)]/[\Delta\tilde{\nu}^{\mathrm{ML}}(\mathrm{RT}) - \Delta\tilde{\nu}^{\mathrm{bubble}}(\mathrm{RT})]$. The solid lines display the strain calculated with the same approach, but starting from the fitting curves displayed in panel **b**.

2.2 Strain estimation for InSe

The InSe flake deposited on the TMD bubbles should undergo a relatively modest strain, since it is just laid on the bubbles without any constraint acting on the InSe flake (specifically, at the bubbles' edges). Instead, the MLs forming the bubbles are indeed subjected to a strong pressure acting inside the bubbles and their border is clamped to the TMD flake underneath by van der Waals forces [19].

To verify whether some the deposition process of InSe on the bubbles induces some strain, we relied on photoluminescence (PL) measurements. Figure 2.3 shows a survey of the difference between the InSe exciton energies measured by room temperature PL on the HS-bubbles (IN) and regions out of the bubbles (OUT). The same analysis was done both for InSe deposited on TMD bubbles and for InSe deposited on hBN bubbles. An average red-shift (as indicated by the negative sign) of 13.7 ± 6.9 meV is found for InSe on the TMD bubbles, while a redshift of 26.0 ± 8.0 meV is found for InSe on the hBN bubbles. The redshift indicates the presence of tensile strain. In fact, the InSe bandgap shrinks upon application of tensile strain to a rate, whose value may range from about 100 meV/% up to about 200 meV/% [21]. The literature-averaged shift rate of the InSe bandgap upon application of total tensile strain is about 150 meV/%. Therefore, the average red-shift of 13.6 ± 6.9 meV of the IN with respect to the OUT regions would correspond to a total tensile strain of about 0.09 %, which would correspond to a biaxial strain of 0.045%. In the case of InSe deposited on hBN the shift is twice as large and the estimated biaxial strain thus amounts to about 0.09 %. This result is particularly interesting if we consider that the aspect ratio (*i.e.*, the ratio between height and radius) of hBN bubbles is smaller (about 0.11) than that of MoS_2 and WS_2 bubbles (about 0.165) [19]. This suggests that InSe adapts better to the flake in the case of flatter bubbles, resulting in a better strain transfer despite the smaller aspect ratio. It's worth noticing that the strain estimated for InSe on hBN bubbles is also consistent with previous experiments in which WS_2 and WSe_2 monolayers were deposited on hBN bubbles, resulting in a small strain transfer with values between 0.06 and 0.10 %. [22].

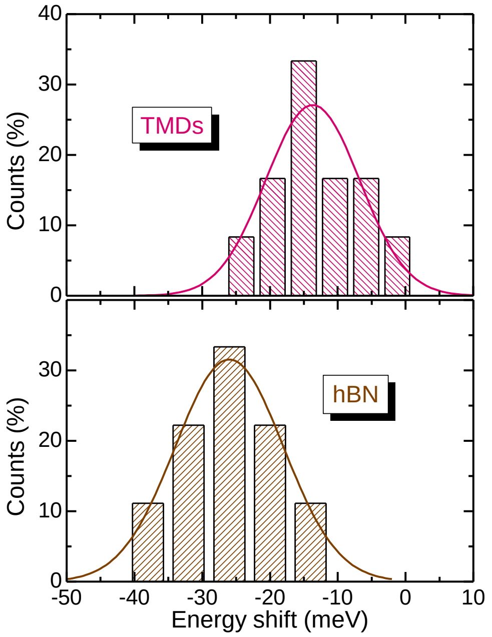

Figure 2.3: Histograms of the energy shifts between the InSe PL band measured at room-temperature on the heterostructured bubbles (IN regions) and outside of the bubbles (OUT regions).

Based on these considerations, we derive that a small (yet non-null) amount of strain is acting on the InSe layer. Such strain is not expected to affect the PL enhancement we observed experimentally. To support this claim, in Figure 2.4 we display the enhancement measured on 6 InSe/WS_2 heterostructured bubbles on a same flake versus the energy shift of the PL band. The shift is also translated into strain (as discussed above) in the top x axis. Indeed, no correlation is found between the enhancement and the shift, thus supporting the hypothesis that such small strain values of InSe do not play significant role in activating the enhancement. This claim is further supported by density functional theory calculations, as discussed later in Supporting Note 7.

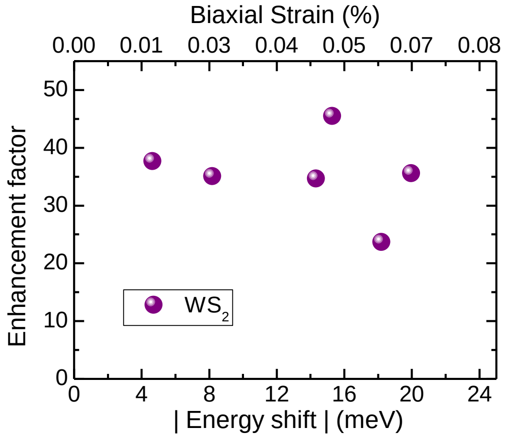

Figure 2.4: Enhancement factor measured on 6 heterostructured bubbled on a same WS_2 flake, as a function of the absolute value of the redshift displayed by the bubbles.

Supporting Note 3. Photoluminescence studies of heterostructured bubbles *vs* temperature and *vs* photogenerated carrier density

3.1 PL *vs* T

To verify how the PL enhancement observed in our selectively strained HSs evolves with temperature, we performed PL measurements as a function of T on two hBN/InSe/MoS_2 HS-bubbles. The AFM image of the selected HS-bubbles is shown in Fig. 3.1. The wrinkled structure on top of the bubbles is due to the hBN capping. Fig. 3.2**a** shows the result of μ-PL measurements

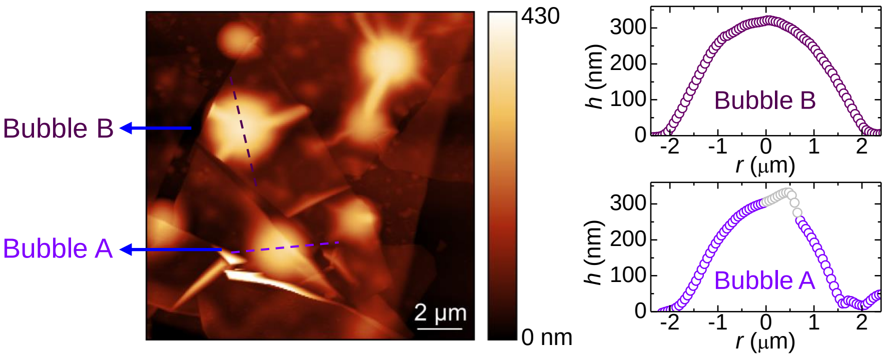

Figure 3.1: Atomic force microscope image of two heterostructured bubbles studied by photoluminescence measurements *vs* temperature. RT AFM image of the two hBN/InSe/MoS_2 HS-bubbles investigated by PL measurements as a function of T. On the right, the profiles corresponding to the dashed lines in the 2D AFM image are shown. The gray data for bubble A highlight the presence of a wrinkle in the hBN flake that caps the heterostructure.

performed on the two HS-bubbles, in the energy region of the InSe PL emission. For comparison, the PL measurements acquired on a flat region of the sample (Out), where InSe lies over the MoS_2 bulk flake, are also displayed. A sizable shift of the PL band with T is evident for both HS-bubbles, as also highlighted by the quantitative analysis of the peak energy presented in panel **b**. To the contrary, the PL band in the Out region undergoes minor changes in energy, with the peak energy displaying an S-shape behaviour when decreasing T. Such an S-shape behaviour was already reported in the literature for few-layer-thick InSe, and was attributed to the dominant presence of defect-related recombination [23]. Noticeably, a rather different behaviour is observed for the HS-bubbles with respect to the Out region. Such behaviour resembles that typical of bandgap-related recombination, and can be explained by hypothesising that the strain-activated charge carrier tunnelling from the TMD to InSe favours the creation and recombination of electron-hole pairs from the band edges of InSe, rather than from defect states. As for the PL intensity, Fig. 3.2**c**, top panel, shows instead the PL peak intensity registered for the two bubbles and for the flat region outside, as a function of temperature. Indeed, in all cases, the PL signal intensity increases with decreasing T. The PL signal collected from the HS bubbles is more intense (by one-to-two orders of magnitude) than the signal acquired in the Out region all over the temperature range, as also highlighted in panel **d**, where the enhancement factor for the two HS-bubbles is displayed. No clear trend with temperature is observed, while the enhancement shows some oscillations with T. This can be attributed to the combined effect of the bandgap variation of both InSe and MoS_2 with T, and of some strain variations occurring with T, likely resulting in a better electronic coupling between the constituent materials of the HS for some specific temperature values.

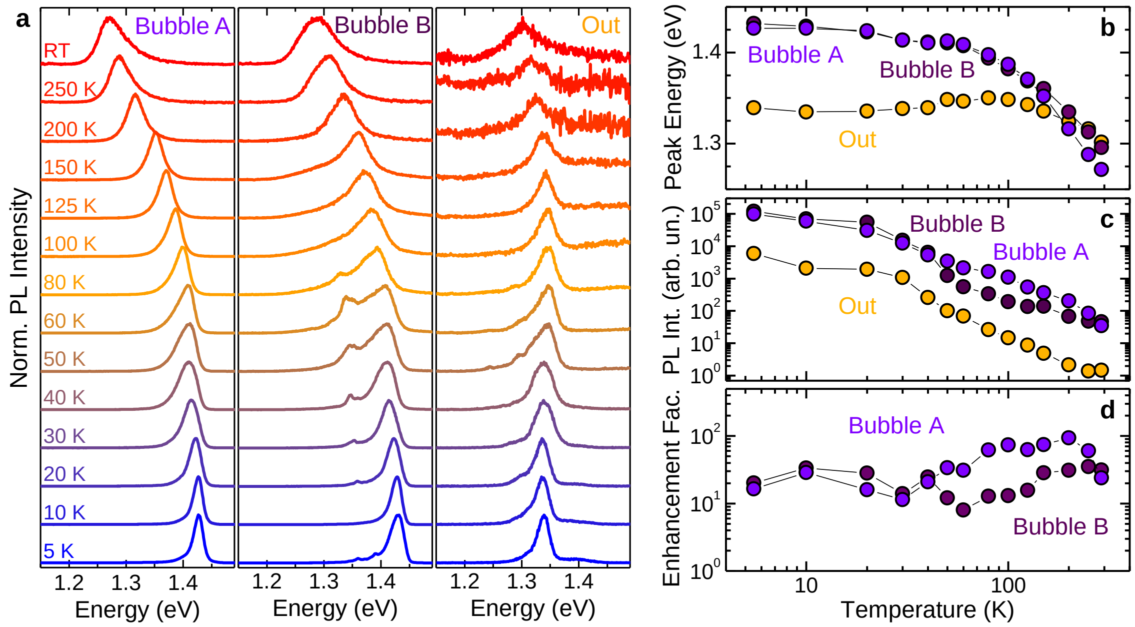

Figure 3.2: Photoluminescence emission of heterostructured bubbles *vs* temperature. **a** PL spectra of two hBN/InSe/MoS_2 HS-bubbles (whose AFM image is shown in Fig. 3.1) and PL signal acquired on a flat region (Out) at different temperatures, in the region of the InSe signal, stacked by y-offset. **b** Peak energy of the InSe band for the two HS-bubbles and the Out region, as a function of T. **c** PL peak intensity of signal from the two HS-bubbles and from the Out region. **d** PL enhancement factor calculated for the two HS-bubbles from the intensity values of panel **c**.

3.2 PL *vs* P_{exc}

In order to further elucidate the blueshift observed at low temperatures and the role played by the coupling of InSe with the strained TMD, we performed further micro-PL measurements as a function of the excitation laser power at 5 K. Such measurements were performed on a hBN/InSe/WS_2 HS-bubble. Specifically, we selected a HS bubble in which the InSe layer is subjected to a nearly null residual strain of about 0.03% (as determined by room-temperature PL measurements showing a blackshift of 8 meV). In turn, this allows us to make a meaningful comparison between the PL lineshapes of the different regions avoiding the effect of the (small) strain acting on the InSe deposited on the TMD bubble.

Low temperature (5 K) measurements as a function of the excitation laser power P_{exc} were performed on the InSe flake region deposited on a WS_2 bubble (*i.e.,* IN) and on the adjacent InSe flat area deposited on the TMD bulk-like flake (*i.e.* OUT). Figure 3.3 **a** shows that with increasing the laser power P_{exc} (namely, the photogenerated carrier density) the InSe emission band undergoes a clear blue-shift in both cases. Concomitantly, the low-energy tail apparent especially for the lowest P_{exc} values becomes less pronounced. These behaviors are characteristics of recombination processes dominated by localised states positioned at energies lower than the free exciton band. Typically, in semiconductors these states originate from crystalline disorder, such as defect states and/or compositional clustering [24]. The presence of these states manifests itself also in a T-dependence of the PL peak energy, which does not follow the usual monotonic decrease when T is increased, but exhibits a plateau-like or an S-shaped behavior [24] like the ones we show in Figure 3.2 and previously reported in 9-layer thick InSe [23]. The spectral weight of the defect states on the PL lineshape is diminished by either raising the lattice temperature, which ionises the defect-localised carriers, and/or the density of photo-generated carriers, which saturates the (finite-density) defect states.

In light of these observations, the blue-shift of the InSe band in the IN region with respect

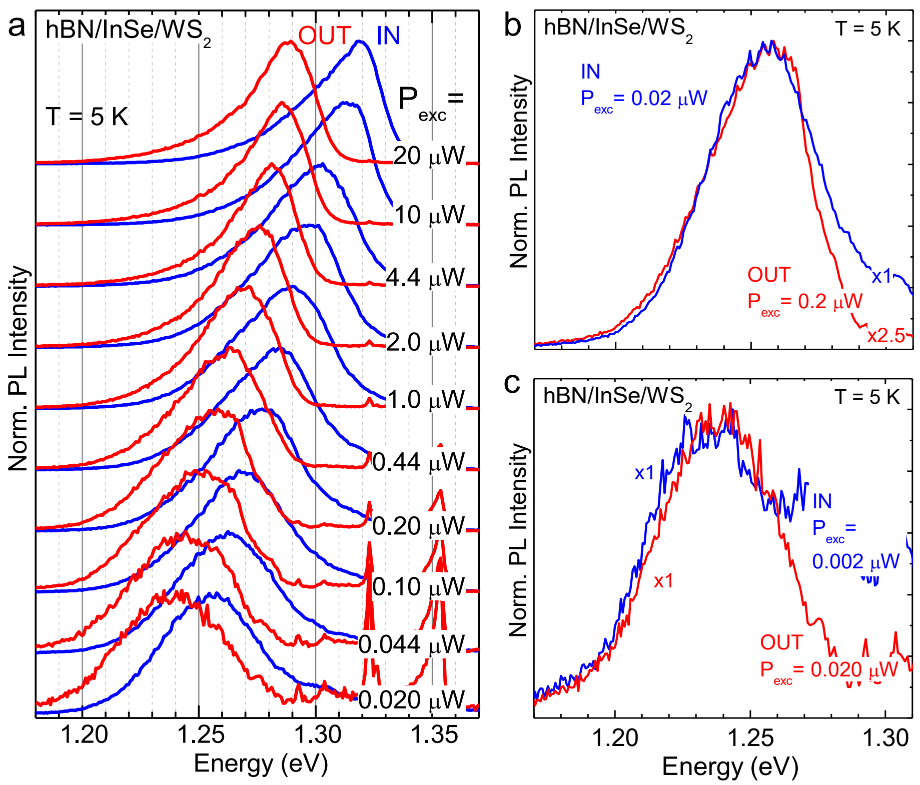

Figure 3.3: **a** T=5 K normalised μ-PL spectra recorded as a function of the laser power P_{exc} on a hBN/InSe/WS_2 HS. IN and OUT refer to spectra recorded on top of the HS-bubble and on the adjacent flat region, respectively. The narrow lines observed on the high-energy side of the OUT region originate from the WS_2 bulk layer. **b** Normalised spectra from part **a** for IN and OUT regions recorded at different P_{exc} values (relative PL normalisation factors are shown). The spectra are compared in order to emphasize that similar peak energies and lineshapes can be observed provided that different P_{exc}s are employed (specifically a factor 10 less in the IN region with respect to the OUT region). **c** The same as **b** for a P_{exc} value for IN as small as 2.0 nW (relative PL normalisation factors are shown).

to the OUT region can be ascribed to an effectively larger photo-generated carrier density in the IN regions of the HSs. As discussed in the manuscript, the origin of this larger density of photo-generated carrier in the IN regions is caused by the extra-carriers that tunnel from the TMD bubble toward the InSe layer deposited on top, together forming a type-I HS. As a matter of fact, we note that the peak energy and lineshape of the PL band of the InSe emission in the IN regions overlap that of the OUT regions for P_{exc} values that are an order of magnitude smaller in the former (IN) with respect to the latter (OUT) case. This is shown in Figure 3.3 **b** and **c** for two different P_{exc}s. In other words, due to the extra carries that tunnel from the TMD to InSe, in the IN InSe regions a factor-10 lower photo-generated carrier density provokes a localised state saturation of the same extent as that achievable in the OUT InSe regions.

Supporting Note 4. Photoluminescence studies of several control samples

To verify that the emission enhancement observed in our HS-bubbles is due to the effect of strain solely, and not (i) to the fact that the InSe layer is coupled to a MS_2 ML, (ii) to a Fabry-Perót effect caused by the presence of a transparent H_2-filled bubble underneath, or (iii) to exciton-dipole orientation effects related to the curved nature of our HSs, we prepared the following control samples: (i) planar (unstrained) hBN/InSe/MS_2-ML HSs deposited atop of a bulk hBN flake; (ii)-(iii) HS-bubbles where the bubble is made of hBN instead of MS_2 (the hBN bubble was still created by hydrogen-ion irradiation [2]). The μ-PL spectra of the control samples are shown

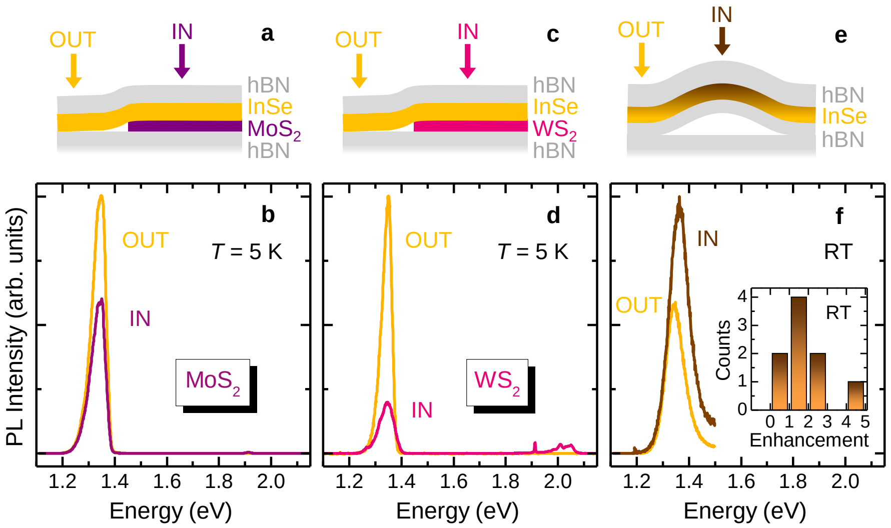

Figure 4.1: Photoluminescence measurements of control heterostructures. **a-d** Sketches and PL spectra of planar (unstrained) hBN/InSe/MS_2 HSs (MoS_2 in panels **a-b**, WS_2 in panels **c-d**). A comparison with the PL spectra in the regions where the TMD is not present and thus only InSe is present is provided. **e-f** Sketch (**e**) and PL spectrum (**f**) of an hBN/InSe/hBN HS-bubble (*i.e.*, where the TMD bubble is replaced by a hBN bubble). The PL spectrum acquired on the bubble is compared with the PL spectrum right outside the bubble. Inset: Statistical analysis of the enhancement factor (I_{IN}/I_{OUT}) measured in several hBN/InSe/hBN HS-bubbles at RT, with 8L- and 9L-thick InSe flakes. For the samples of panels **b**, **d** and **f** a 8L-thick InSe was used.

in Fig. 4.1. For case (i), Figs. 4.1**a-d** show HSs formed by depositing a 8L-InSe sample on a planar (*i.e.*, strain-free) MoS_2 (**a-b**) or WS_2 (**c-d**) ML. A decrease of the InSe PL signal is observed in both cases (like in Ref. [25]), contrary to the PL enhancement observed in the strained case. Analogous PL reductions are also found in similar HSs with different InSe thickness, as shown later. For cases (ii)-(iii), Figs. 4.1**e-f** show the instance of a 8L-InSe on a hBN bubble, where, instead, a PL increase by about a factor 2 is found. Similar measurements were performed on other hBN/InSe/hBN HS-bubbles, and I_{IN}/I_{OUT} values between 0.3 and 4.4 were observed, see inset of Fig. 4.1**f**. These moderate signal reductions or –more frequently– enhancements can be likely attributed to interference effects and/or to the effect of dipole selection rules related to light polarisation. Considering the second case, we note that the bubbles have a softly curved structure with a height-to-radius ratio of ≈ 0.11 for hBN [2] and ≈ 0.165 for MoS_2 and WS_2 [19] (corresponding to an inclination of at most 13° and 20°, respectively, at the very edge of the bubbles), while the laser is polarised parallel to the substrate plane. Given the out-of-plane dipole of InSe [26, 27, 28], this can lead to an increased light absorption in the inclined InSe layer with respect to the planar situation. PL enhancements of a factor of 2-3 were indeed proved for multilayer ($N > 3$) InSe flakes bent on pillars with an inclination up to 9° [29] and for thick

InSe flakes in the shape of ridges with a 10°-30° deflection [30]. Our control measurements on hBN bubbles (Fig. 4.1**f**) show consistent results. Such enhancements are indeed much smaller than those found for our strained TMD-based HSs.

Fig. 4.2 shows the results of PL measurements performed on further unstrained HSs formed by depositing InSe flakes of various thicknesses —in between 6 and 8 layers— on planar (*i.e.*, strain-free) MoS_2 (**a-b**) or WS_2 (**c-d**) MLs. A decrease of the InSe PL signal in the HS region (IN) with respect to the bare InSe flake (OUT) is observed in all cases, contrary to the PL enhancement observed in the strained case.

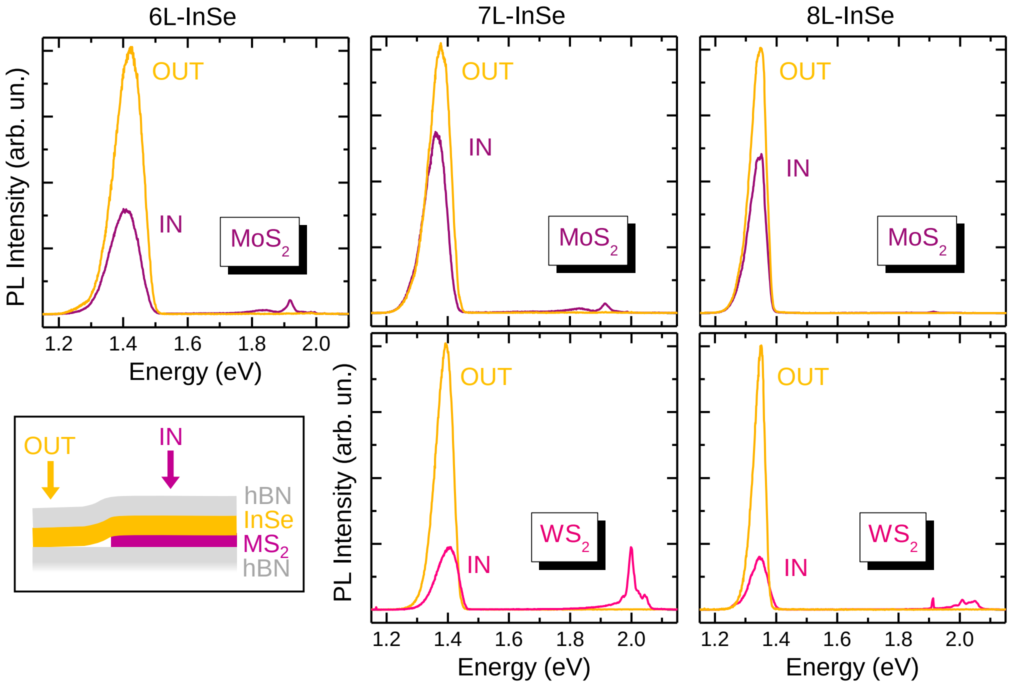

Figure 4.2: Photoluminescence measurements of control planar heterostructures. PL spectra of planar (*i.e.*, where the MS_2 ML is not strained) hBN/InSe/MS_2 HS (IN, see sketch at the bottom-left), and comparison with the PL spectra in the regions where the TMD is not present and thus only InSe is present (OUT, see sketch at the bottom-left). Different InSe flake thicknesses, in between 6 and 8 layers, are considered.

Supporting Note 5. Photoluminescence excitation studies of an unstrained hBN/InSe/MoS_2 heterostructure

Fig. 5.1 shows the PLE spectrum of the hBN/6L-InSe/MoS_2 planar HS whose PL spectrum is shown in Fig. 4.2. The PLE spectrum was acquired by monitoring the PL signal of InSe while the excitation laser energy was changed. Indeed, at variance with the PLE spectrum of the strained HS bubble (shown in Fig. 4 of the main text), in this case no clear resonance is observed, suggesting that no charge transfer from the MoS_2 ML to the InSe flake occurs. This result is consistent with the absence of PL enhancement observed in our planar HSs (see Supporting Note 4) and in Ref. [25].

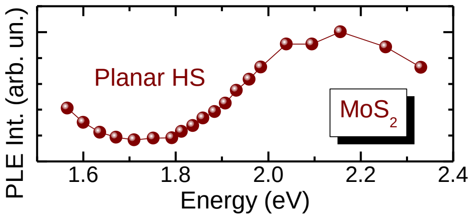

Figure 5.1: Photoluminescence excitation measurement of a planar heterostructure. PLE spectrum of the hBN/6L-InSe/MoS_2 planar HS of Fig. 4.2.

Supporting Note 6. DFT simulations of explicit InSe/MoS_2 interfaces.

We built explicit HSs between 6L-InSe and ML-MoS_2 with a sulphur vacancy. While 6L-InSe was unstrained, the TMD ML was studied both at 0% and at 2% biaxial tensile strain. Side and top view of both HSs are shown in Figure 6.1.

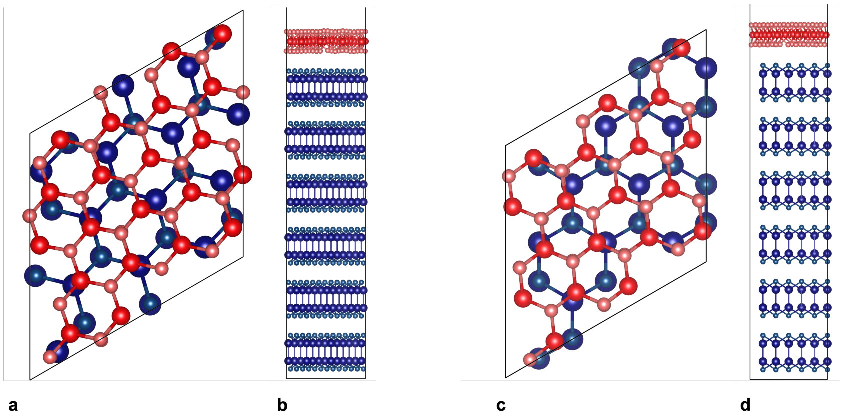

Figure 6.1: Optimised geometries of explicit HSs made of 6L-InSe and MoS_2 ML with sulphur vacancy. **a-b**: top and side view of the HS with the unstrained MoS_2 ML, respectively. **c-d**: top and side view of the HS with MoS_2 ML at 2% biaxial tensile strain, respectively. Large dark red and smaller light red spheres mark Mo and S atoms, respectively. Large dark blue and smaller light blue spheres mark In and Se atoms, respectively.

The unstrained and strained HSs count 3504 and 3224 electrons, respectively. All calculations, from geometry optimisation to band structure and projected density of states (pDOS) evaluation were performed with the GGA-based rev-vdW-DF2 functional [14] without spin-orbit coupling. Band structures and k-resolved pDOS (k-pDOS) are shown for k-points in the Brillouin zone of the relative HS.

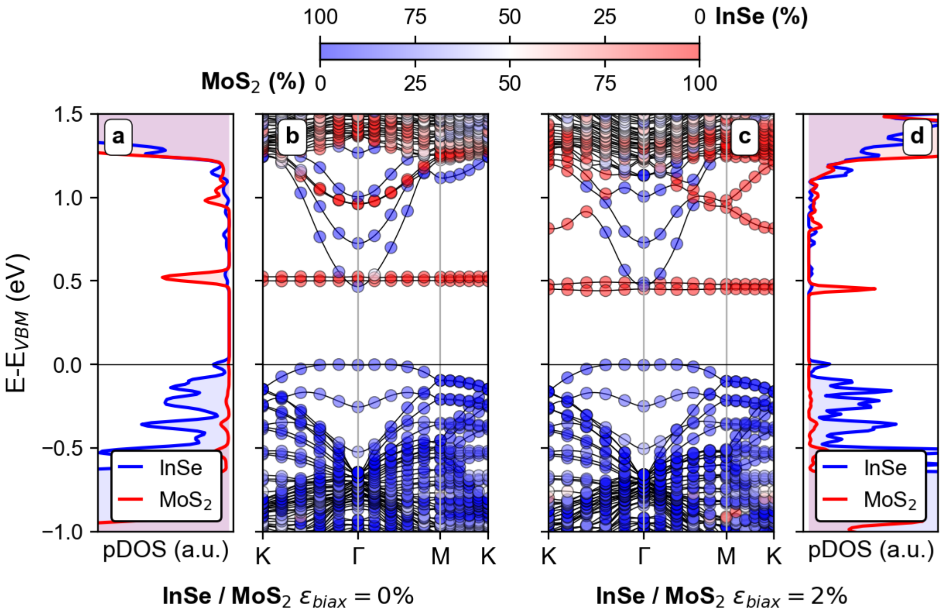

Figure 6.2: Band structures, k-pDOS and pDOS of the HSs made of 6L-InSe and MoS_2 ML with a sulphur vacancy. **a-b** Unstrained MoS_2 ML case. **c-d** 2% strained MoS_2 ML case.

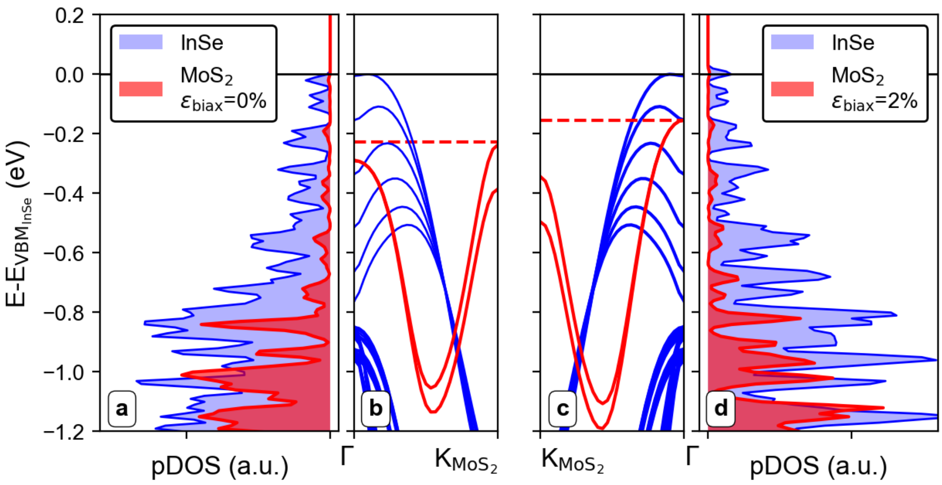

Figure 6.3: pDOS of the explicit HSs with unstrained, (panel **a**), and strained (panel **d**) ML-MoS_2. In panels **b** and **c** we report the relative valence band alignment as predicted from GGA calculations via the method in Ref. [11].

In Figure 6.2 we report the band structures with superimposed the k-resolved density of states projected on the atomic obitals of 6L-InSe and ML-MoS_2. Red and blue dots corresponds to electronic states composed of MoS_2 or InSe atomic orbitals, respectively. Intermediate shades mark hybridization. Notice that in Figure 6.2**b**, the K/K' points of MoS_2 are folded to the Γ point of the HS Brillouin zone.

In Figure 6.3 we report a detail of the pDOS of 6L-InSe and ML-MoS_2 in the explicit HSs for $\varepsilon_{\text{biax}} = 0\%$ of MoS_2, in panel **a** and $\varepsilon_{\text{biax}} = 2\%$ in **d**. We compare the pDOS obtained from the full simulations of the HSs to the aligned band structures of both materials (panels **b** and **c**), in their unit cells. The band alignment in panels **b** and **c** is obtained from PBE calculations, independently of the explicit HSs simulations, and following the method in Ref. [11], used for the aligment of the HSE band structures in the main text.

The comparison between the reconstructed alignments in panels **b** and **c** and the relative pDOSs in panels **a** and **b**, respectively, further confirms that the top valence band of MoS_2 at the Γ point aligns with the topmost valence bands of 6L-InSe. In particular, the VBM of the strained MoS_2 monolayer, at Γ, shifts closer to the VBM of InSe.

Supporting Note 7. DFT simulations of strained InSe.

In Fig. 7.1 we report the DFT calculations of the electronic bandstructures of 6L-InSe with applied biaxial strain $\varepsilon_{\text{biax}} = 0\%, 0.1\%$ (*i.e.*, close to the experimental value), 1%, 2%. The

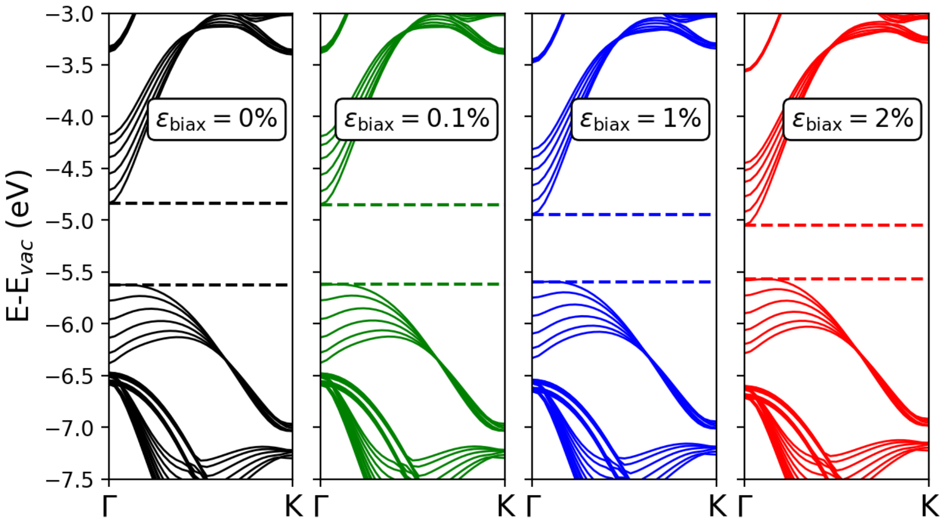

Figure 7.1: Evolution of the electronic bandstructures of 6L-InSe under applied biaxial strain. The bandstructures are aligned to the vacuum level.

bandstructures are aligned to the vacuum level for comparison. Tensile strain results in a reduction of the electronic bandgap, mainly due to the downshift in energy of the CBM at Γ. On the contrary, the VBM of the strained samples remains aligned to the VBM of the unstrained one. This shows that the discussed InSe/TMD band alignment is preserved even with strained InSe and the proposed charge transfer mechanism would not be affected by the presence of strain.

References

[1] D. Tedeschi, E. Blundo, M. Felici, G. Pettinari, B. Liu, T. Yildrim, E. Petroni, C. Zhang, Y. Zhu, S. Sennato, Y. Lu, and A. Polimeni, *Controlled Micro/Nanodome Formation in Proton-Irradiated Bulk Transition-Metal Dichalcogenides*, Adv. Mater. **31**, 1903795 (2019).

[2] E. Blundo, A. Surrente, D. Spirito, G. Pettinari, T. Yildirim, C. A. Chavarin, L. Baldassarre, M. Felici, and A. Polimeni, *Vibrational properties in highly strained hexagonal boron nitride bubbles*, Nano Lett. **22**, 4, 1525 (2022).

[3] N. Bauer and J. Y. Beach, *A Comparison of the Ionization Cross Sections of H_2 and D_2*, J. Chem. Phys. **16**, 7, 837 (1948).

[4] S. Hu, Lozada-Hidalgo, F. Wang, Mishchenko, F. Schedin, R. Nair, Boukhalov, M. Katsnelson, R. Dryfe, I. Grigorieva, H. Wu, and A. Geim, *Proton transport through one-atom-thick crystals*, Nature **516**, 227–230 (2014).

[5] M. Seel and R. Pandey, *Proton and hydrogen transport through two-dimensional monolayers*, 2D Mater. **3**, 2, 025004 (2016).

[6] P. Giannozzi, S. Baroni, N. Bonini, M. Calandra, R. Car, C. Cavazzoni, D. Ceresoli, G. L. Chiarotti, M. Cococcioni, I. Dabo, A. D. Corso, S. de Gironcoli, S. Fabris, G. Fratesi, R. Gebauer, U. Gerstmann, C. Gougoussis, A. Kokalj, M. Lazzeri, L. Martin-Samos, N. Marzari, F. Mauri, R. Mazzarello, S. Paolini, A. Pasquarello, L. Paulatto, C. Sbraccia, S. Scandolo, G. Sclauzero, A. P. Seitsonen, A. Smogunov, P. Umari, and R. M. Wentzcovitch, *QUANTUM ESPRESSO: a modular and open-source software project for quantum simulations of materials*, J. Phys. Condens. Mat. **21**, 39, 395502 (2009).

[7] P. Giannozzi, O. Andreussi, T. Brumme, O. Bunau, M. B. Nardelli, M. Calandra, R. Car, C. Cavazzoni, D. Ceresoli, M. Cococcioni, N. Colonna, I. Carnimeo, A. D. Corso, S. de Gironcoli, P. Delugas, R. A. DiStasio, A. Ferretti, A. Floris, G. Fratesi, G. Fugallo, R. Gebauer, U. Gerstmann, F. Giustino, T. Gorni, J. Jia, M. Kawamura, H.-Y. Ko, A. Kokalj, E. Küçükbenli, M. Lazzeri, M. Marsili, N. Marzari, F. Mauri, N. L. Nguyen, H.-V. Nguyen, A. O. de-la Roza, L. Paulatto, S. Poncé, D. Rocca, R. Sabatini, B. Santra, M. Schlipf, A. P. Seitsonen, A. Smogunov, I. Timrov, T. Thonhauser, P. Umari, N. Vast, X. Wu, and S. Baroni, *Advanced capabilities for materials modelling with Quantum ESPRESSO*, J. Phys. Condens. Mat. **29**, 46, 465901 (2017).

[8] M. van Setten, M. Giantomassi, E. Bousquet, M. Verstraete, D. Hamann, X. Gonze, and G.-M. Rignanese, *The PseudoDojo: Training and grading a 85 element optimized norm-conserving pseudopotential table*, Comput. Phys. Commun. **226**, 39–54 (2018).

[9] J. P. Perdew, K. Burke, and Y. Wang, *Generalized gradient approximation for the exchange-correlation hole of a many-electron system*, Phys. Rev. B **54**, 16533–16539 (1996).

[10] J. Heyd, G. E. Scuseria, and M. Ernzerhof, *Hybrid functionals based on a screened Coulomb potential*, J. Chem. Phys. **118**, 18, 8207–8215 (2003).

[11] Y. Hinuma, A. Grüneis, G. Kresse, and F. Oba, *Band alignment of semiconductors from density-functional theory and many-body perturbation theory*, Phys. Rev. B **90**, 155405 (2014).

[12] G. W. Mudd, A. Patanè, Z. R. Kudrynskyi, M. W. Fay, O. Makarovsky, L. Eaves, Z. D. Kovalyuk, V. Zólyomi, and V. Falko, *Quantum confined acceptors and donors in InSe nanosheets*, Appl. Phys. Lett. **105**, 22, 221909 (2014).

[13] P. Lazić, *CellMatch: Combining two unit cells into a common supercell with minimal strain*, Comput. Phys. Commun. **197**, 324–334 (2015).

[14] I. Hamada, *van der Waals density functional made accurate*, Phys. Rev. B **89**, 121103 (2014).

[15] S. Cianci, E. Blundo, F. Tuzi, G. Pettinari, K. Olkowska-Pucko, E. Parmenopoulou, D. B. Peeters, A. Miriametro, T. Taniguchi, K. Watanabe, A. Babinski, M. R. Molas, M. Felici, and A. Polimeni, *Spatially Controlled Single Photon Emitters in hBN-Capped WS_2 Domes*, Adv. Optical Mater. **11**, 2202953 (2023).

[16] J. Sun, J. Li, Y. Jiang, X. Ma, Z. Tan, and G. Zhufu, *Key Construction Technology and Monitoring of Long-Span Steel Box Tied Arch Bridge*, Int. J. Steel Struct. **23**, 191 (2023).

[17] P. Klemens, *Anharmonic decay of optical phonons*, Phys. Rev. **148,**, 845 (1966).

[18] M. Balkanski, R. Wallis, and E. Haro, *Anharmonic effects in light scattering due to optical phonons in silicon*, Phys. Rev. B **28,**, 1928 (1983).

[19] E. Blundo, T. Yildirim, G. Pettinari, and A. Polimeni, *Experimental Adhesion Energy in van der Waals Crystals and Heterostructures from Atomically Thin Bubbles*, Phys. Rev. Lett. **127**, 4, 046101 (2021).

[20] C. Postmus, J. Ferraro, and S. Mitra, *Pressure dependence of infrared eigenfrequencies of KCl and KBr*, Phys. Rev. **174,**, 983 (1968).

[21] E. Blundo, E. Cappelluti, M. Felici, G. Pettinari, and A. Polimeni, *Strain-tuning of the electronic, optical, and vibrational properties of two-dimensional crystals*, Appl. Phys. Rev. **8**, 021318 (2021).

[22] E. Blundo, P. E. Faria Junior, A. Surrente, G. Pettinari, M. A. Prosnikov, K. Olkowska-Pucko, K. Zollner, T. Woźniak, A. Chaves, T. Kazimierczuk, M. Felici, A. Babiński, M. R. Molas, P. C. M. Christianen, and J. F. A. Polimeni, *Strain-Induced Exciton Hybridization in WS_2 Monolayers Unveiled by Zeeman-Splitting Measurements*, Phys. Rev. Lett. **129**, 067402 (2022).

[23] T. Venanzi, H. Arora, S. Winnerl, A. Pashkin, P. Chava, A. Patanè, Z. D. Kovalyuk, Z. R. Kudrynskyi, K. Watanabe, T. Taniguchi, A. Erbe, M. Helm, and H. Schneider, *Photoluminescence dynamics in few-layer InSe*, Phys. Rev. Mater. **4**, 044001 (2020).

[24] A. Polimeni, M. Capizzi, M. ans Geddo, M. Fischer, M. Reinhardt, and A. Forchel, *Effect of temperature on the optical properties of (InGa)(AsN)/GaAs single quantum wells*, Appl. Phys. Lett. **77**, 2870 (2000).

[25] Z.-Y. Sun, Y. Li, B. Xu, H. Chen, P. Wang, S.-X. Zhao, L. Yang, B. Gao, X.-M. Dou, B.-Q. Sun, L. Zhen, , and C.-Y. Xu, *Tailoring the Energy Funneling across the Interface in InSe/MoS_2 Heterostructures by Electrostatic Gating and Strain Engineering*, Adv. Optical Mater. **9**, 4, 2100438 (2021).

[26] D. A. Bandurin, A. V. Tyurnina, , G. L. Yu, A. Mishchenko, V. Zólyomi, S. V. Morozov, R. K. Kumar, R. V. Gorbachev, Z. R. Kudrynskyi, S. Pezzini, Z. D. Kovalyuk, U. Zeitler, K. S. Novoselov, A. Patanè, L. Eaves, I. V. Grigorieva, V. I. Fal'ko, A. K. Geim, and Y. Cao, *High electron mobility, quantum Hall effect and anomalous optical response in atomically thin InSe*, Nat. Nanotechnol. **12**, 223 (2017).

[27] G. W. Mudd, M. R. Molas, X. Chen, V. Zólyomi, K. Nogajewski, Z. R. Kudrynskyi, Z. D. Kovalyuk, G. Yusa, O. Makarovsky, L. Eaves, M. Potemski, V. I. Fal'ko, and A. Patanè, *The direct-to-indirect band gap crossover in two-dimensional van der Waals indium selenide crystals*, Sci. Rep. **6**, 39619 (2016).

[28] M. Brotons-Gisbert, R. Proux, R. Picard, D. Andres-Penares, A. Branny, A. Molina-Sánchez, J. F. Sánchez-Royo, and B. D. Gerardot, *Out-of-plane orientation of luminescent excitons in two-dimensional indium selenide*, Nat. Commun. **10**, 3913 (2019).

[29] D. Mazumder, J. Xie, Z. R. Kudrynskyi, X. Wang, O. Makarovsky, M. A. Bhuiyan, H. Kim, T.-Y. Chang, D. L. Huffaker, Z. D. Kovalyuk, L. Zhang, and A. Patané, *Enhanced Optical Emission from 2D InSe Bent onto Si-Pillars*, Adv. Optical Mater. **8**, 2000828 (2020).

[30] Y. Li, T. Wang, H. Wang, Z. Li, Y. Chen, D. West, R. Sankar, R. K. Ulaganathan, F. Chou, C. Wetzel, C. Y. Xu, S. Zhang, and S. F. Shi, *Enhanced light emission from the ridge of two-dimensional InSe flakes*, Nano Lett. **18**, 8, 5078—5084 (2018).
